# Perceived barriers to the adoption of active surveillance in low-risk prostate cancer: a qualitative analysis of community and academic urologists

**DOI:** 10.1186/s12885-021-08386-3

**Published:** 2021-05-31

**Authors:** Shellie D. Ellis, Soohyun Hwang, Emily Morrow, Kim S. Kimminau, Kelly Goonan, Laurie Petty, Edward Ellerbeck, J. Brantley Thrasher

**Affiliations:** 1 Department of Population Health, School of Medicine, University of Kansas, Kansas City, KS USA; 2 Department of Health Policy and Management, School of Public Health, University of North Carolina Chapel Hill, 135 Dauer Drive, 1101 McGavran-Greenberg Hall, Chapel Hill, NC 27599-7411 USA; 3 Department of Sociology, University of Kansas, Kansas City, KS USA; 4 Department of Family Medicine, School of Medicine, University of Kansas, Kansas City, KS USA; 5 Independent Researcher/Consultant/Scientific Writer, Greensboro, NC USA; 6 American Board of Urology, Charlottesville, VA USA

**Keywords:** Active surveillance, Prostate cancer, Adoption, Barriers, Low-risk disease

## Abstract

**Background:**

Clinical practice guidelines recommend active surveillance as the preferred treatment option for low-risk prostate cancer, but only a minority of eligible men receive active surveillance, and practice variation is substantial. The aim of this study is to describe barriers to urologists’ recommendation of active surveillance in low-risk prostate cancer and explore variation of barriers by setting.

**Methods:**

We conducted semi-structured interviews among 22 practicing urologists, evenly distributed between academic and community practice. We coded barriers to active surveillance according to a conceptual model of determinants of treatment quality to identify potential opportunities for intervention.

**Results:**

Community and academic urologists were generally in agreement on factors influencing active surveillance. Urologists perceived patient-level factors to have the greatest influence on recommendations, particularly tumor pathology, patient age, and judgements about the patient’s ability to adhere to follow-up protocols. They also noted cross-cutting clinical barriers, including concerns about the adequacy of biopsy samples, inconsistent protocols to guide active surveillance, and side effects of biopsy procedures. Urologists had differing opinions on the impact of environmental factors, such as financial disincentives and fear of litigation.

**Conclusions:**

Despite national and international recommendations, both academic and community urologists note a variety of barriers to implementing active surveillance in low risk prostate cancer. These barriers will need to be specifically addressed in efforts to help urologists offer active surveillance more consistently.

## Background

Active surveillance (AS) is one of three guideline-concordant management strategies for men diagnosed with low-risk, localized prostate cancer [1]. Delaying aggressive treatment until defined signs of disease progression have occurred has been a nationally-endorsed treatment strategy for nearly two decades [2, 3], and the focus of efforts to promote its appropriate use since 2011 [4]. With evidence accumulating that AS is safe over periods of six to 10 years among men with low-risk disease [5, 6], there is a growing consensus that it is not merely an evidence-based option for low risk disease, but rather the preferred management strategy [7, 8]. Consensus was formalized in 2016, when the American Society for Clinical Oncology endorsed Canadian guidelines stating that AS should be the preferred option in low-risk disease [8].

Despite consistent recommendation of AS, generally defined as regular PSA testing and annual prostate biopsies to monitor for signs of disease progression and initiate curative therapy, use of this management option has been low. As few as 10% of men newly eligible for AS received it in the previous decade [4, 9–13]. Recent estimates suggest use of AS increased substantially (up to 49% of men with low-risk disease in some areas); however, variation in individual urology practices’ use is substantial, ranging from 8 to 64% [12, 14–19]. Understanding this variation is essential for designing targeted interventions to address underuse.

Reasons for the underuse of AS are incompletely understood. Low rates have been ascribed to patients’ lack of acceptance. However, one study reports that AS is not *offered* as a treatment option to one-third of localized prostate cancer patients [18], and a substantial body of work describing treatment decision making in the context of low-risk prostate cancer has consistently shown that patient preferences are *not* incorporated into treatment selection [17–20]. Because urologists’ recommendations may weigh heavily in prostate cancer patients’ treatment decisions [15, 21–26], exploring factors that influence urologists’ recommendations for AS is needed. Reimbursement, physician factors, [27], and practice setting may influence the care that is delivered [28, 29]. However, few studies have comprehensively investigated the physician, organizational, and policy factors and treatment characteristics that may shape urologists’ beliefs about the appropriateness of AS [12, 30, 31]. We sought to 1) identify a comprehensive array of physician-perceived barriers to the offer of AS, and 2) explore variability in experience by the urologists’ practice setting (academic or community), including the potentially disparate financial pressures, patient populations, and organizational supports for following treatment protocols.

## Methods

**Fig. 1:**
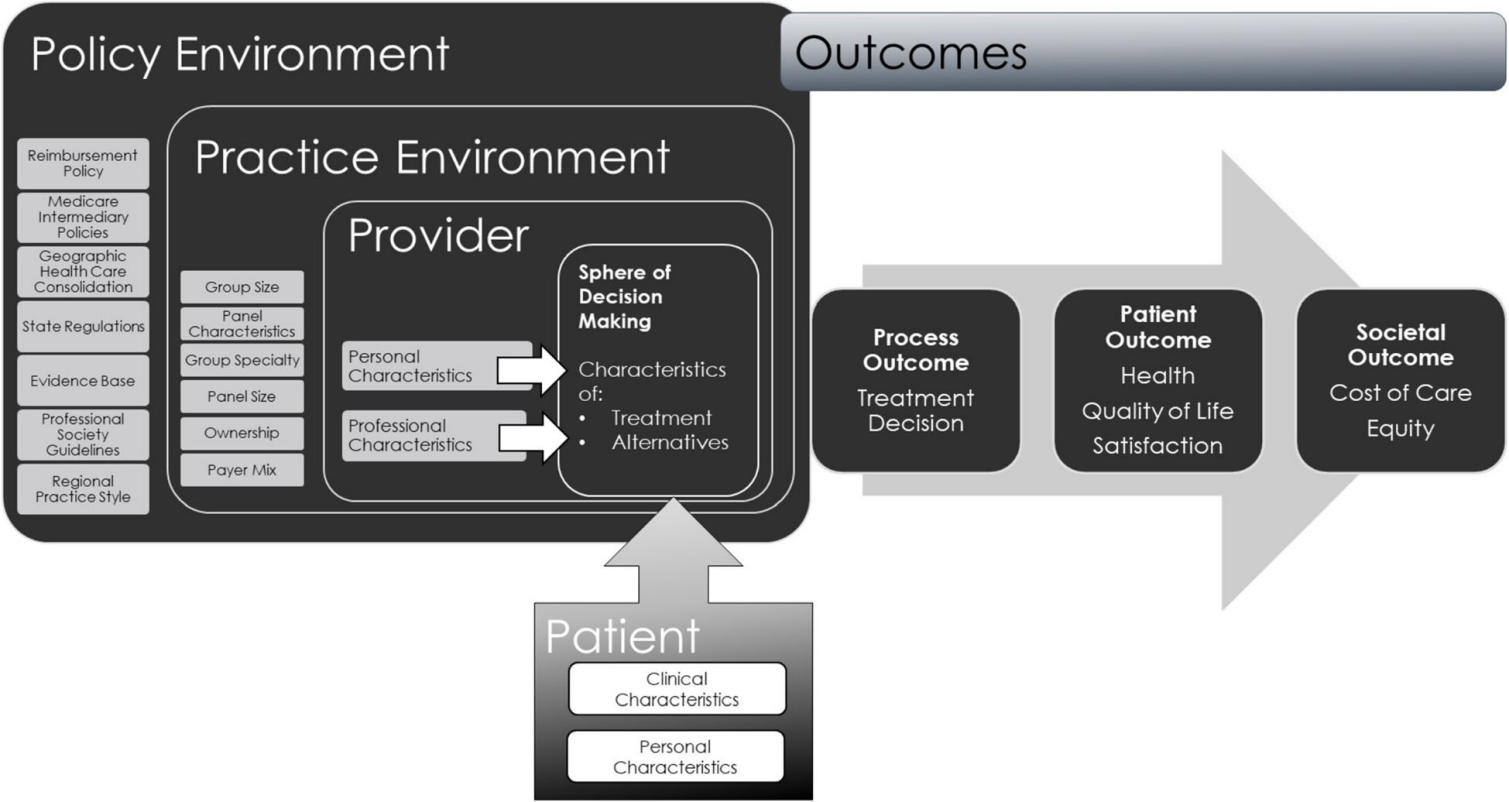
Potential domains of influence on physician recommendations

We conducted a cross-sectional retrospective study comprised of in-depth, qualitative interviews with practicing urologists. The conceptual model underlying this research follows from previous work on localized prostate cancer treatment delivery [29, 32] and mirrors a behavioral model of clinician responses to incentives, which incorporates economic theory [33] to conceptualize how reimbursement context may influence physicians to induce demand for health services [34, 35]. Recognizing that additional macro- and micro-level factors affect treatment decisions, the model derives the concept of predisposing and enabling factors from Andersen and Aday’s *Behavioral Model of Health Service Use* and reconceptualizes them from the healthcare providers’ perspective [36, 37] (Fig. 1**).** The model suggests that, at the patient level, physicians consider each patient’s tumor and clinical characteristics, as well as patients’ personal preferences and specific circumstances. Nonetheless, provider- [38], practice- [39–41], and policy-level [34] factors also directly or indirectly influence treatment recommendations and decision-making that ultimately influence both the patient outcome (e.g., quality of life) and societal outcomes (e.g., cost of care).

### Interview guide development

We developed the interview guide based on our conceptual model and ethnographic interview methods [42]. We pilot tested it to assess participant burden and ensure elicitation of sensitive topics. We assessed tolerance for interview length among a convenience sample of urologists, identifying a 20-min stated tolerance. We pilot-tested the initial interview guide among four urology residents to assess length and whether urologists would discuss non-clinical aspects of care. All pilot participants provided responses to all questions and reported no adverse reactions to potentially sensitive questions. Because the interview length exceeded the 20-min goal, we analyzed pilot transcripts to eliminate duplicative questions and elicitation of routine practice procedures. We restructured the interview guide to be more open-ended with less specific probing and to discriminate between active treatment and surveillance, rather than asking about individual treatment modalities. Questions described as “difficult” were retained to elicit potential disagreement and conditional decision-making rules from them. Ultimately, we identified five essential questions to prioritize in a brief, scheduled interview, and embedded them in a more robust interview guide to allow additional opportunities to explore participant-identified priorities as time allowed.

The resulting interview guide [43] asked participants about their clinic organization and flow; treatments typically offered to low-risk prostate cancer patients; ideal patients for AS; exceptions to, concerns about, and consequences of offering AS; and how other doctors in their social and referral networks responded to their treatment decisions. Probes focused on anticipated barriers to AS, such as biopsy side effects, practice burden, and financial disincentives.

### Recruitment

We used a multi-modal recruitment strategy [44] to invite urologists to participate. The strategy included 1) borrowing the professional networks of clinical colleagues [44]; 2) directly asking attendees at the meeting site; and 3) utilizing the snowball technique (i.e., allowing recruited participants to recommend other potential participants) [45]. Participants received a $300 gift card for participation.

### Data collection

Two interviewers trained in ethnographic methods conducted 22 interviews between May and September 2015. Sixteen interviews were conducted in-person during the American Urological Association (AUA) annual meetings in New Orleans. Six were conducted after the meeting by phone. Interviewers followed the interview guide, ensuring the five essential questions were posed, and used probes to explore topics not broached by participants. All interviews were audio-recorded and transcribed verbatim.

### Coding

Prior to coding in ATLAS.ti [46], two investigators (Ellis and Morrow) developed a codebook of anticipated themes based on the conceptual model. We used the codebook to conduct template analysis [47] and also allowed for themes or subthemes to emerge from the data [48]. We further identified whether the determinant was a barrier to the AS recommendation. To ensure consistency, we double coded a portion of the transcripts and compared code interpretation and application. Disagreements were reconciled by consensus.

### Analysis

We assessed the sample responses for saturation [49] in two steps. First, we assessed participant characteristics among the initial sample of interviews completed at the AUA meeting. We identified that we had recruited few rural urologists, no women urologists, and no African-American urologists. We subsequently recruited urologists representing each of these demographic groups and practice locations. After coding was complete, the new interviews were evaluated for thematic saturation by comparing responses of the targeted participants to those of original participants to see if new themes emerged. We summarized prevalent barriers within the domains of influence, characterizing them both by the number of participants who mentioned them and the number of mentions across all interviews. Finally, we explored differences by practice setting (academic vs. community) in the number of participants describing a barrier and content of participants’ responses. We selected illustrative quotes for presentation and edited them to remove extraneous vernacular for ease of reading.

## Results

Offers to participate were extended to 37 urologists. We recruited 15 participants through the professional networks of four academic urologists and one rural general surgeon; we recruited four participants via direct request at the AUA meeting; and three participants recruited an additional participant each. Combined efforts resulted in 22 completed interviews (60% participation rate). Thirteen of the 15 urologists who did not participate did not respond to email requests; one declined; and one agreed but did not complete the interview. All non-responders were academic urologists. No new barriers or themes were identified in the second round of five additional interviews, meeting the a priori criteria for data saturation. Participants practiced in 11 states, representing each of the four major census regions of the country (Table 1). Urologists’ practices were evenly divided between academic and community settings. Community practices included urban and rural practices, and one privately-owned intensity-modulated radiation therapy (IMRT) center.

**Table 1:** Demographic Characteristics of Participating Urologists

	Study Sample (***N*** = 22)	No. (%)
Sex	Male	21 (95%)
Observed Race/Ethnicity	Caucasian	18 (88%)
Trainee	Yes	3 (14%)
Region	Midwest	11 (50%)
	South	8 (36%)
	West	2 (9%)
	Northeast	1 (5%)
Practice Site	Academic	11 (50%)
	Community	11 (50%)
Recruitment Source	Borrowed Network	15 (68%)
	Direct Ask	4 (18%)
	Snowball	3 (14%)
Interview Type	In-person	16 (73%)
	Phone	6 (27%)

### Relative weight of non-clinical factors

**Fig. 2:**
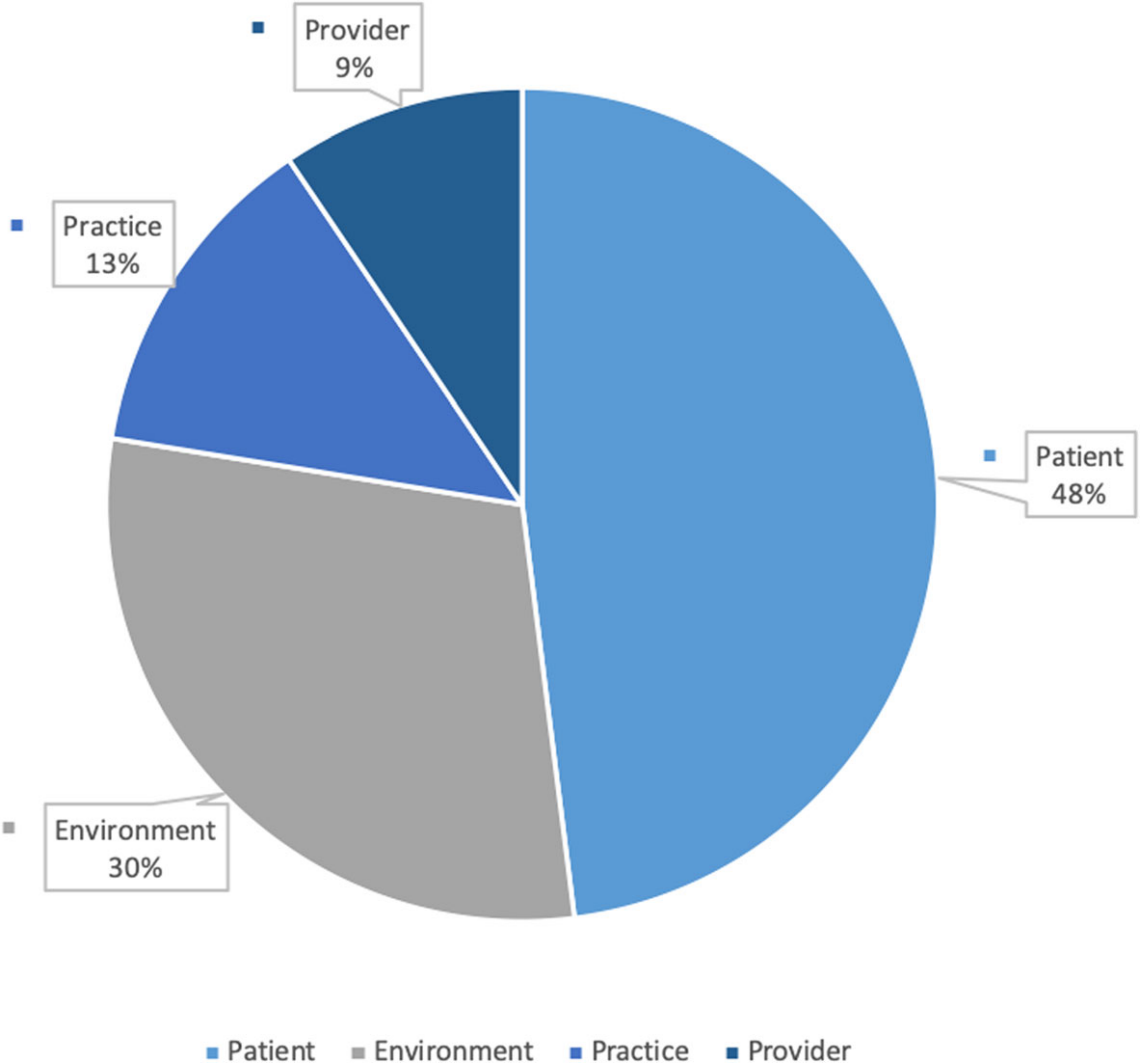
Treatment decision making domains

Urologists addressed a wide range of potential influences specified in the conceptual model: 16 of 17 original domains were identified as contextually or directly relevant to making treatment decisions. Urologists reported treatment recommendations were influenced most heavily (48%) by the clinical and personal characteristics of the patient (Fig. 2). Provider characteristics, practice characteristics, and the environmental context represented 9, 13, and 30% of coded text segments, respectively. Although clinical and non-clinical factors were described as potential barriers, a high concentration of the discussion centered on the treatment options themselves. Urologists considered AS relative to the side effects, potential for cure, and delivery convenience of competing treatment strategies.

### Clinical barriers

#### Tumor pathology

All participants described using clinical classification criteria to assess the appropriateness of AS. Gleason score was the primary criterion discussed (cited in all interviews). Other clinical criteria were mentioned less consistently as decision criteria: volume of cores (*n* = 14), PSA level (*n* = 12), number of cancerous cores (*n* = 11), stage (*n* = 6), and PSA density (*n* = 2, both community urologists).

Thresholds for considering AS appropriate varied. All urologists indicated offering AS to men with Gleason 6 disease. Two urologists (one academic and one community) mentioned some concern even for this low-risk group. The academic urologist described himself as conservative with AS and cited reservations about its use in patients with 2 + 4 disease, who he was concerned may have occult disease. The community urologist indicated he would only feel comfortable offering it if the man had limited life expectancy. Fewer participants (*n* = 9) described AS as appropriate in patients with intermediate risk disease. Most expressed caution about the strategy for men in this group, suggesting they would weigh the clinical criteria (considering the number of criteria, which put the patient in the intermediate risk group), and limit the recommendation to men with 3 + 4 disease (as compared to a single severe pathology) or only offer it to older men.

#### Age

Participants considered additional clinical criteria that impacted their recommendation. Among them, the most prevalent clinical criterion shaping AS recommendation was patient age, described as relevant by almost half of the urologists, with concerns (primarily from academic urologists) centering around the appropriateness of AS for young patients.

“If they’re older, and they meet all the criteria, then I think everyone is in agreement with active surveillance for them, but it’s probably that younger age group. It’s hard to make that decision of committing them to treatment at a young age and dealing with the side effects versus watching them and potentially missing a cancer that could progress.” – Academic Urologist.

Academic urologists in particular expressed discomfort recommending AS to young patients due to the lack of data on long-term outcomes and unstudied trade-offs between AS and early aggressive treatment.

“… what’s worse? an active intervention [at a young age] when we can preserve as much of the healthy tissue as we can, be very aggressive about nerve sparing, and get the best results in that patient population, or is it best to subject them to five, eight, ten, 15 years of annual biopsies because we still don’t understand how to monitor progression...” – Academic Urologist.

Urologists wanted to minimize side effects but had differing views on how to do this particularly for young men. For some urologists, young patients were precisely the patients for whom they wanted to delay clinical intervention because of the potential side effects of treatment. Conversely, others felt young patients were the most likely to recover well from aggressive therapy and avoid its side effects.

#### Other clinical criteria

Other clinical characteristics were mentioned infrequently. Two urologists (one academic and one community) indicated concerns about the appropriateness of AS for African-American men. Two other urologists (one academic and one community) explained they would be unlikely to recommend AS to patients experiencing voiding issues. In their understanding, voiding issues would not be resolved through AS, but could be through surgical treatment.

### Patient characteristics

Approximately one-third of urologists mentioned needing to feel comfortable with the patient’s ability to adhere to the AS protocol before proceeding with AS.

“Normally we would’ve done active surveillance but … we’re not sure exactly how reliable he’s going to be.” – Academic Urologist.

These concerns were heightened with young, otherwise healthy patients. Due to the greater length of time they would spend on the protocol, urologists had to trust in the patient’s ability to follow up to consider recommending AS to them. Determination of potential adherence was largely subjective.

“How educated they are plays a big role in it and just how well they interact with you when you’re doing the discussion because if they’re asking a lot of questions, they’re probably going to stay on top of things. If they are just sitting there ‘well doc, whatever you tell me,’ they may not be quite as likely to follow up and do what is said.” – Academic Urologist.

Sometimes assessment was based on factors such as distance to care, availability of social support, uncontrolled comorbid disease, or a history of missed appointments.

“I have a guy … he had pretty high chances of having prostate cancer, and put off his diagnostic biopsy for two years, and missed three or four appointments during that time. He’s not a guy that I would be excited about putting on active surveillance, even if he had disease parameters that were favorable.” – Community Urologist.

Some urologists cited other factors they felt predicted adherence, such as the patient’s education level, inability to afford transportation, and patients being designated unreliable by the primary care physician.

### Diagnosis/treatment limitations

#### Biopsy limitations

The most frequently reported category of barrier was related to current technology available to diagnose and treat prostate cancer. More than half of the urologists expressed uneasiness about offering AS because the prostate biopsy used for diagnosis is based on tissue sampling and leaves opportunity to miss an aggressive cancer. Urologists talked about this limitation intensely (30 mentions of it by 12 physicians). The inability to “see” the cancer was problematic as was the reality that prostate biopsies rely on only sampling small portions of the entire tissue for signs of disease.

“And in the back of your mind a biopsy is just that. It’s supposed to be representative of what’s going on in the total gland, but it’s a small sample of it and so you don’t know what’s around those areas that you missed.” – Community Urologist.

“How accurate is that biopsy? Based on data through the years, we get it right probably two-thirds of the time and we get it wrong roughly one-third of the time so, are we making the right decision?” – Community Urologist.

Kidney cancer, you can see the mass. Testes cancer, you can see the mass. Bladder cancer, most of the time you can see the mass. Prostate, you don’t. It’s a normal gland staring at you through the ultrasound probe, and yet it’s hiding in there. And that’s what’s so distressing. You can’t define it. You can’t visually wrap your brain around it. All you can do is stick the needle in a bunch of times and kind of get a general sense of where the heck it is. Very frustrating.” – Academic Urologist.

#### AS protocol limitations

The lack of a standard evidence-based surveillance protocol was the second treatment concern.

“There’s a lot of variation in the protocol. There are several published protocol series that range in terms of frequency of biopsy, PSA and prostate exam. I think that we don’t know the best way to do it and I think probably it will get more lenient over time, in terms of how many repeat biopsies people are getting.”

– Academic Urologist.

This sentiment was echoed by community urologists as well, with one noting the subjectivity of the follow-up protocol and uncertainty in the treatment itself due to the lack of an evidence-based AS protocol. Uncertainty about the follow-up protocol was heightened in the treatment of younger men for whom urologists felt the greatest uncertainty in managing prostate cancer with AS.

“I’m worried that we don’t know -- we have lots of relatively short-term studies when it comes to prostate cancer [treated with] AS, but we don’t have 25 years down the road.” – Community Urologist.

#### Biopsy side effects

Urologists described patients’ concerns about biopsy; however, urologists themselves had few concerns about the side effects of biopsy.

“Well you know that a lot of times repetitive biopsy is part of it, and that makes -- it’s not so much a barrier to offering it. It’s a barrier to getting it accepted.”

– Community Urologist.

Several urologists acknowledged there were potential side effects. They did not perceive these side effects to deter their own decision-making, but believed they could dissuade patients from continuing an AS protocol if they occurred.

“For people who had a bad experience with the biopsy, either it’s painful, they had a lot of bleeding, or painful urination afterward. Some of those patients would say, ‘I don’t want another biopsy.’ So, they might be bad candidates for active surveillance, in terms of the logistics of actually doing it. In terms of the complications, they’re pretty uncommon. I think if someone had sepsis after a biopsy, then they may need more aggressive procedural antibiotics or something like that to prevent that from happening again; or like a rectal swab biopsy. Some way to help limit their risk of sepsis. In terms of the biopsy complications, most patients tolerate it fairly well, I think. We put a lot of lidocaine -- we use lidocaine injection and use lidocaine jelly into the rectal vault and there’s variation on how people tolerate it. But, I think for the most part, most people do okay. Most people do fine.” – Academic Urologist.

### Policy and practice environment

#### Practice environment

Almost one-third of urologists discussed environmental factors affecting their decision, but the specific factors influencing the AS offer were diffuse. Intensive direct-to-consumer marketing of new surgical or radiation modalities (mentioned by four participants) sometimes made it difficult for urologists to persuade patients to accept AS, but was not acknowledged as having direct influence on any urologist’s own treatment recommendations. Although some academic urologists believed that fear of litigation would make their private practice peers more likely to pursue possibly unnecessary or unduly risky aggressive therapies, fear of litigation was mentioned in our study by only two community urologists and it was not uniformly seen as a barrier to AS. One community urologist reported fear he would be sued if he recommended AS and the patient later developed a more aggressive cancer and poor outcome.

“I think the biggest concern is litigious... that you’re going to miss something and it’s going to slip through the cracks and by the time you get to surgery or by the time you get to radiation that you’re going to have a failure.” – Community Urologist.

We also found the opposite -i.e., concern that recommending surgery to someone, who was also eligible for AS, could result in litigation if there was a negative outcome (e.g., side effects) from the more aggressive treatment.

“It’s a highly litigious world … we do have active surveillance or even watchful waiting, and sometimes those options are better for patients, but they still elect a treatment that could cause them to have problems. [Like] radiation therapy on an older patient who has a really big prostate, he’s already having urinary symptoms and he elects radiation, and in the back of your mind you’re saying this guy’s going to be coming in with worsening symptoms and maybe we’ll do some procedure on him, maybe he’ll get some scar tissue -- just kind of thinking on the worse lines of side effects of treatment.” – Community Urologist.

#### Reimbursement

Academic urologists assumed reimbursement incentives to offer aggressive treatment would hinder community urologists’ willingness to recommend AS: “I think [my community colleagues] see AS as an intrusion on their business, frankly.” However, comments from community urologists did not support this assumption. Several community urologists indicated high patient volume insulated them from financial pressures.

“I don’t think the [doctors in my practice] consider the revenue aspect of it. … We have enough work, so I don’t think it matters.” – Community Urologist.

Although community urologists were cognizant of differential reimbursement between surgery and AS, they indicated they were not aware of the overall financial impact of the difference.

“[AS] probably affects our revenue … Well, if you were to have a radical prostatectomy, the charge is higher than the upfront charge for surveillance. But the surveillance probably brings in more revenue over time than radio therapy does, for us. So I mean, it’s up and down both, but I don’t know how it is overall.” – Community Urologist.

### Internal practice factors

#### Clinical practice impact

Most urologists from both the academic organizations and the community insisted there was no direct impact of AS to their clinical workflow. Since there is no new role/responsibility or any other substantial changes for the clinicians, this was not perceived as a barrier for adopting AS into their practice for managing patients with low-risk prostate cancer.

“If anything, it’s just when do you have them follow-up. It’s just another patient that fits into the slot that I don’t think they have any extra needs or anything like that, that disturbs workflow or has extra constraints on the clinic or needs there.” – Academic Physician.

One urologist did not acknowledge this as a barrier to AS but did note the increased follow-up would add more volume of procedures and potentially influence clinical practice.

“… They may continue with us, but the active surveillance, you’re just, your follow up population is growing exponentially because you’re seeing them initially at three months to get a few PSA’s, and then at six months, and then the volume of biopsies goes way up. So it does fill your practice a lot more.” – Academic Physician.

The impact of AS on the practice was little discussed possibly because in most practices low-risk prostate cancer is not highly prevalent:

“Prostate cancer’s such a small portion of my practice anyway, that if I’ve got 20 percent of 10 percent of my population on an active surveillance protocol, that’s not a real big issue for me.” – Community Urologist.

## Discussion

This study is the first to our knowledge to comprehensively assess a broad range of potential barriers that influence U.S. urologists’ decision-making in low-risk prostate cancer. We found that both community and academic urologists in our sample were generally accepting of AS. Both groups reported using guideline-recommended criteria to assign treatments for low-risk disease, with some variability for intermediate-risk disease. Similar to other studies, we found clinical factors, such as Gleason score and patient age, were associated with urologists’ recommendation [20].

Urologists perceived decision-making to be strongly influenced by clinical criteria and patient preference [50] and self-report few individual physician- and practice-level barriers to recommending AS. Of note, external influences reported elsewhere to be potential barriers to the offer of AS (e.g., medico-legal constraints, financial incentives) were not identified by participants in our sample [27]. Instead, other factors related to treatment technology (diagnostic inaccuracy and the lack of evidence supporting AS protocols) [4, 51, 52], incomplete evidence regarding treatment for young men, and subjective predictions of future adherence were frequently discussed and hold potential to constrain recommendation of AS as a management strategy.

Our finding that physicians perceive rapidly evolving and non-standardized follow-up protocols as a barrier to the adoption of AS has also been reported previously [53]. Research is underway to clarify biopsy and surveillance protocols, which may reduce uncertainty in selecting and following patients on AS [5, 54–56], particularly for young men [54]. Prospective research on appropriate recommendations for patients who are at higher risk for prostate cancer death, such as African American men, also may be warranted. Emerging research on the risk of reclassification during AS among African American men [57] may partially address this. In the meantime, consistency across guidelines on the best approaches from trusted sources, such as the American Urological Association [58] and the National Comprehensive Cancer Network [51] may limit confusion.

The uncertainty of prostate biopsy, a recommended component of the AS follow-up protocol, weighed heavily in the active surveillance recommendation and many new technologies are in development to increase the ability to differentiate slow growing and aggressive tumors. Interestingly, physicians did not express concerns about the side effects of biopsy, although there is a growing body of popular and empirical literature addressing the complications of biopsies [59, 60], including infection, erectile dysfunction, and voiding dysfunction associated with multiple transrectal ultrasound biopsies [60, 61].

Urologists’ concerns about patients’ ability to adhere to AS protocols has also been previously reported [62]. Although urologists reported mentioning all treatment options to patients they defined as clinically appropriate, they altered their recommendations based on their judgment of what was appropriate for each patient. The level of enthusiasm in which a physician discusses treatment options has been demonstrated to affect patients’ uptake of options [22, 63]. However, physicians’ predictions of a patient’s ability to adhere to a treatment protocol have been shown to be unreliable, whether made solely on subjective judgments of patient characteristics or based on patient behavior evidence in the medical record [64, 65]. A potential solution is implementation of shared decision making, which may be underused in urology practice [66, 67]. Rather than deciding subjectively who might have trouble adhering to an AS protocol, the urologist or care team could instead elicit values from the fully informed patient to arrive at treatment selection together. **S**hared decision-making practices may further help urologists clarify the values of very young (and very old) patients to ensure preferences are identified and reflected in treatment decisions [15].

Despite intensive probing, some barriers we expected to be reported based on prior literature (e.g., urologists’ fear of litigation [68] and financial incentives to provide surgical treatment [68–73]) were not substantiated. Litigation fears supported both aggressive treatment and AS. Our study may have uncovered geographic variability in community standards, although the diverging opinions we identified both occurred in the Southeast US. It is also possible that litigation concerns are used to justify preferred treatment patterns rather than driving treatment preferences. Numerous reports in the research literature and popular press allude to profit motives of urologists in suggesting (over) treatment [69–75]. The urologists in our study were aware of scrutiny over physician-induced demand faced by the specialty. Academic urologists felt above reproach on these matters but made assumptions that their community-based colleagues were more susceptible to financial pressures. However, the community and private practice physicians (including the one working in a private IMRT facility, a care model at the center of controversy over physician-induced demand) described high volume workloads or patient panels not appropriate for AS. Other carefully designed studies that attempt to identify or explain apparent physician induced demand raise doubt that financial incentives alone motivate urologists’ treatment decisions [29, 32, 76, 77]. Because the relationship between potential financial motivations and treatment recommendations is not clear, more work may be needed to carefully untangle the circumstances in which financial motivations may influence care.

Also contrary to our expectation, nearly all participants perceived AS did not disturb existing clinical workflows; rather, it was viewed as a straightforward incorporation into existing clinic time allocated to follow-up appointments. Whether this reflects little real impact of AS protocols in the context of urologists’ heterogenous practice mix and multiple professional roles or poor adherence to existing protocols, we cannot ascertain. Our study did not observe the actual AS protocols followed by the urologists and thus, we cannot know if AS protocols were carried out as intended. Previous research has shown variation in the percentage of patients receiving guideline-concordant AS follow-up care [31, 78], which may be attributable to patient non-adherence in addition to the lack of a standardized follow-up protocol in the practice. More research on the objective impact on clinical practice is warranted.

### Limitations

There are several important limitations to the study. Our study was designed to identify the range of influences on AS recommendation. Although we were able to describe the relative weight of the domains of influence across our small sample of urologists, we did not survey a representative sample of urologists about the importance of these factors in their specific decisions. The sample of 22 urologists was adequate to reach saturation to sufficiently identify a range of perspectives, but our study was not designed to infer that each of these opinions are held by all urologists. Large scale surveys are needed to assess the degree to which these opinions are held, among which subpopulations of urologists. Likewise, we were able to recruit physicians equally from academic and community practices; community physicians represented a variety of private practice models; and no new themes emerged after additional community physicians were included. However, future research should directly compare barriers reported by AS adopters and non-adopters to identify the degree to which these findings are generalizable to all urologists [50].

Social desirability bias may have influenced urologists to indicate stronger support for AS than they may actually offer in practice, and they may have downplayed barriers. Thus, our work may under-represent barriers urologists face, underscoring the importance of further survey research exploring all barriers mentioned. Finally, we interviewed urologists only and did not assess patients’ barriers to AS. Elsewhere, we report urologists’ perceptions of the barriers patients report [62] and others have well described patient barriers [21, 22, 24, 79, 80]. Although patient preferences are vital to treatment decision-making in a preference-sensitive decision, such as prostate cancer treatment, urologists remain the gatekeepers to patients’ treatment selection. Even though urologists themselves perceive patient barriers to be tantamount, patients cannot be resistant to choices about which they have not been informed. Thus, the barriers urologists face must be removed before patient barriers can be fully addressed.

## Conclusions

Physician recommendations are of paramount importance to men faced with making treatment decisions. Despite urologists’ emphasis on clinical features and patient preference, we find that lack of confidence in the treatment technology and some patients’ ability to adhere are of primary concern. Suspected influences, such as fear of being sued and financial incentives to offer alternatives, were less apparent. This deeper understanding of the barriers that urologists perceive to be important can guide future interventions to reduce variation in the offer of AS.

## Data Availability

The datasets generated during the current study are not publicly available due to the confidentiality afforded study participants, but are available in limited, redacted form from the corresponding author on reasonable request.

## Abbreviations

AS: Active Surveillance
AUA: American Urological Association

## References

[1] Network NCC (2010). NCCN Clinical Practice Guidelines in Oncology (NCCN Guidelines™). Practice Guidelines in Oncology.

[2] Bahnson RR, Hanks GE, Huben RP, Kantoff P, Kozlowski JM, Kuettel M, Lange PH, Logothetis C, Pow-Sang JM, Roach M (2000). nccn practice guidelines for prostate cancer. Oncology (Williston Park).

[3] Network NCC (2004). Prostate cancer. NCCN practice guidelines in oncology.

[4] Ganz P, Barry J, Burke W, Col N, Corso P, Dodson E, Hammond M, Kogan B, Lynch C, Newcomer L, Seifter EJ, Tooze JA, Viswanath KV, Wessells H (2011). National Institutes of Health state-of-the-science conference statement: role of active surveillance in the management of men with localized prostate cancer. NIH Consens State Sci Statements.

[5] Yamamoto T, Musunuru B, Vesprini D, Zhang L, Ghanem G, Loblaw A, et al. Metastatic prostate cancer in men initially managed with active surveillance. J Urol. 2015;193(4S). 10.1016/j.juro.2015.02.149. 10.1016/j.juro.2015.11.075 26707510

[6] Tosoian JJ, Mamawala M, Epstein JI, Landis P, Wolf S, Trock BJ, Carter HB (2015). Intermediate and longer-term outcomes from a prospective active-surveillance program for favorable-risk prostate cancer. J Clin Oncol.

[7] Cooperberg MR, Carroll PR (2015). Treatment trends for prostate Cancer--reply. JAMA.

[8] Chen RC, Rumble RB, Loblaw DA, et al. Active Surveillance for the Management of Localized Prostate Cancer (Cancer Care Ontario Guideline): American Society of Clinical Oncology Clinical Practice Guideline Endorsement. J Clin Oncol. 2016;34(18):2182-90. 10.1200/JCO.2015.65.7759. 10.1200/JCO.2015.65.7759 26884580

[9] Hall IJ, Richardson LC (2012). Commentary on the state-of-the-science conference on the role of active surveillance in the management of men with localized prostate cancer. J Natl Cancer Inst Monogr.

[10] Chamie K, Williams SB, Hu JC (2015). Population-based assessment of determining treatments for prostate cancer. JAMA Oncol.

[11] Chamie K, Williams SB, Hershman DL, Wright JD, Nguyen PL, Hu JC (2015). Population-based assessment of determining predictors for quality of prostate cancer surveillance. Cancer.

[12] Cooperberg MR, Carroll PR (2015). Trends in Management for Patients with Localized Prostate Cancer, 1990-2013. JAMA.

[13] Barocas DA, Cowan JE, Smith JA, Carroll PR, Ca PI (2008). What percentage of patients with newly diagnosed carcinoma of the prostate are candidates for surveillance? An analysis of the CaPSURE database. J Urol.

[14] Womble PR, Montie JE, Ye Z, Linsell SM, Lane BR, Miller DC (2015). Michigan urological surgery improvement C: contemporary use of initial active surveillance among men in Michigan with low-risk prostate cancer. Eur Urol.

[15] Scherr KA, Fagerlin A, Hofer T, et al. Physician Recommendations Trump Patient Preferences in Prostate Cancer Treatment Decisions. Med Decis Making. 2017;37(1):56-69. 10.1177/0272989X16662841. 10.1177/0272989X16662841 PMC5587215 27510740

[16] Reamer E, Yang F, Holmes-Rovner M, Liu J, Xu J (2017). Influence of Men's personality and social support on treatment decision-making for localized prostate cancer. Biomed Res Int.

[17] Holmes-Rovner M, Srikanth A, Henry SG, Langford A, Rovner DR, Fagerlin A. Decision aid use during post-biopsy consultations for localized prostate cancer. Health Expect. 2018;21:279–87. https://doi-org.libproxy.lib.unc.edu/10.1111/hex.12613. 10.1111/hex.12613 PMC5750733 28881105

[18] Holmes-Rovner M, Montgomery JS, Rovner DR, Scherer LD, Whitfield J, Kahn VC, Merkle EC, Ubel PA, Fagerlin A (2015). Informed decision making: assessment of the quality of physician communication about prostate Cancer diagnosis and treatment. Med Decis Making.

[19] Adsul P, Wray R, Spradling K, Darwish O, Weaver N, Siddiqui S (2015). Systematic review of decision Aids for newly diagnosed patients with prostate cancer making treatment decisions. J Urol.

[20] Scherr KA, Fagerlin A, Hofer T, Scherer LD, Holmes-Rovner M, Williamson LD, Kahn VC, Montgomery JS, Greene KL, Zhang B, Ubel PA (2017). Physician recommendations trump patient preferences in prostate cancer treatment decisions. Med Decis Making.

[21] Showalter TN, Mishra MV, Bridges JF (2015). Factors that influence patient preferences for prostate cancer management options: a systematic review. Patient Prefer Adherence.

[22] Donovan JL (2012). Presenting treatment options to men with clinically localized prostate cancer: the acceptability of active surveillance/monitoring. J Natl Cancer Inst Monogr.

[23] Sidana A, Hernandez DJ, Feng Z, Partin AW, Trock BJ, Saha S, Epstein JI (2012). Treatment decision-making for localized prostate cancer: what younger men choose and why. Prostate.

[24] Xu J, Neale AV, Dailey RK, Eggly S, Schwartz KL (2012). Patient perspective on watchful waiting/active surveillance for localized prostate cancer. J Am Board Fam Med.

[25] Zeliadt SB, Moinpour CM, Blough DK, Penson DF, Hall IJ, Smith JL, Ekwueme DU, Thompson IM, Keane TE, Ramsey SD (2010). Preliminary treatment considerations among men with newly diagnosed prostate cancer. Am J Manag Care.

[26] Shahinian VB, Kuo YF, Freeman JL, Orihuela E, Goodwin JS (2007). Characteristics of urologists predict the use of androgen deprivation therapy for prostate cancer. J Clin Oncol.

[27] Loeb S, Curnyn C, Fagerlin A, Braithwaite RS, Schwartz MD, Lepor H, Carter HB, Sedlander E (2017). Qualitative study on decision-making by prostate cancer physicians during active surveillance. BJU Int.

[28] Spencer BA, Miller DC, Litwin MS, Ritchey JD, Stewart AK, Dunn RL, Gay EG, Sandler HM, Wei JT (2008). Variations in quality of care for men with early-stage prostate cancer. J Clin Oncol.

[29] Ellis SD, Nielsen ME, Carpenter WR, Jackson GL, Wheeler SB, Liu H, Weinberger M (2015). Gonadotropin-releasing hormone agonist overuse: urologists' response to reimbursement and characteristics associated with persistent overuse. Prostate Cancer Prostatic Dis.

[30] Cooperberg MR, Broering JM, Carroll PR (2010). Time trends and local variation in primary treatment of localized prostate cancer. J Clin Oncol.

[31] Luckenbaugh AN, Auffenberg GB, Hawken SR, Dhir A, Linsell S, Kaul S, et al. Michigan urological surgery improvement C: variation in guideline concordant active surveillance follow-up in diverse urology practices. J Urol. 2016;195(4S). 10.1016/j.juro.2016.02.2528. 10.1016/j.juro.2016.09.071 PMC5315618 27663459

[32] Ellis SD, Chen RC, Dusetzina SB, Wheeler SB, Jackson GL, Nielsen ME, Carpenter WR, Weinberger M (2016). Are small reimbursement changes enough to change cancer care? reimbursement variation in prostate cancer treatment. J Oncol Pract.

[33] Frolich A, Talavera JA, Broadhead P, Dudley RA (2007). A behavioral model of clinician responses to incentives to improve quality. Health Policy.

[34] Feldstein PJ (2007). Health policy issues: an economic perspective.

[35] Rice TH (1983). The impact of changing medicare reimbursement rates on physician-induced demand. Med Care.

[36] Aday LA, Andersen R (1974). A framework for the study of access to medical care. Health Serv Res.

[37] Andersen RM (1995). Revisiting the behavioral model and access to medical care: does it matter?. J Health Soc Behav.

[38] Golden BR, Sloan FA, Sloan FA, Kasper H (2008). Physician pay for performance: alternative perspectives. Incentives and Choice in Health Care.

[39] Strand H, Parker D (2012). Effects of multidisciplinary models of care for adult pre-dialysis patients with chronic kidney disease: a systematic review. Int J Evid Based Healthc.

[40] Kim MM, Barnato AE, Angus DC, Fleisher LA, Kahn JM (2010). The effect of multidisciplinary care teams on intensive care unit mortality. Arch Intern Med.

[41] Fleissig A, Jenkins V, Catt S, Fallowfield L (2006). Multidisciplinary teams in cancer care: are they effective in the UK?. Lancet Oncol.

[42] Spradley J (1979). The ethnographic interview Fort Worth: Harcourt.

[43] Brooks JV, Ellis SD, Morrow E, Kimminau KS, Thrasher JB (2018). Patient factors that influence how physicians discuss active surveillance with low-risk prostate cancer patients: a qualitative study. Am J Mens Health.

[44] Ellis SD, Bertoni AG, Bonds DE, Clinch CR, Balasubramanyam A, Blackwell C, Chen H, Lischke M, Goff DC (2007). Value of recruitment strategies used in a primary care practice-based trial. Contemp Clin Trials.

[45] Valente TW, Davis RL (1999). Accelerating the diffusion of innovations using opinion leaders. Ann Am Acad Polit Ss.

[46] Scientific Software: atlas.ti: the knowledge workbench. Ver 6.2. ATLAS.ti Scientific Software Development GmbH. 2011.

[47] Symon G, Cassell C (1998). Qualitative methods and analysis in organizational research: a practical guide.

[48] Creswell JW (1998). Qualitative inquiry and research design: choosing among five traditions.

[49] Creswell JW (2014). Research design: qualitative, quantitative and mixed methods approaches 4th Ed.

[50] Kim SP, Gross CP, Nguyen PL, Smaldone MC, Shah ND, Karnes RJ, Thompson RH, Han LC, Yu JB, Trinh QD, Ziegenfuss JY, Sun M, Tilburt JC (2014). Perceptions of active surveillance and treatment recommendations for low-risk prostate Cancer: results from a National Survey of radiation oncologists and urologists. Med Care.

[51] NCC N, National Comprehensive Cancer Network (2016). Prostate Cancer. NCCN Clinical Practice Guidelines in Oncology (NCCN Guidelines).

[52] Roth JA, Ramsey SD, Carlson JJ (2015). Cost-effectiveness of a biopsy-based 8-protein prostate cancer prognostic assay to optimize treatment decision making in Gleason 3 + 3 and 3 + 4 early stage prostate cancer. Oncologist.

[53] Pang K, Fitch M, Ouellet V, Chevalier S, Drachenberg DE, Finelli A, Lattouf JB, So A, Sutcliffe S, Tanguay S, Saad F, Mes-Masson AM (2018). Describing perspectives of health care professionals on active surveillance for the management of prostate cancer. BMC Health Serv Res.

[54] Klotz L, Vesprini D, Sethukavalan P, Jethava V, Zhang L, Jain S, Yamamoto T, Mamedov A, Loblaw A (2015). Long-term follow-up of a large active surveillance cohort of patients with prostate cancer. J Clin Oncol.

[55] Cher ML, Dhir A, Auffenberg GB, Linsell S, Gao Y, Rosenberg B, et al. Appropriateness criteria for active surveillance of prostate cancer. J Urol. 2016;195(4S). 10.1016/j.juro.2016.02.2527. 10.1016/j.juro.2016.07.005 27422298

[56] Cooperberg MR (2015). Long-term active surveillance for prostate cancer: answers and questions. J Clin Oncol.

[57] Schenk JM, Newcomb LF, Zheng Y, Faino AV, Zhu K, Nyame YA, Brooks JD, Carroll PR, Cooperberg MR, Dash A, Filson CP, Gleave ME, Liss M, Martin FM, Morgan TM, Nelson PS, Thompson IM, Wagner AA, Lin DW (2020). African American race is not associated with risk of reclassification during active surveillance: results from the canary prostate cancer active surveillance study. J Urol.

[58] Association AU (2017). Prostate Cancer. Clinically Localized Prostate Cancer: AUA/ASTRO/SUO Guideline (2017).

[59] Gettman MT (2012). Better understanding of minimizing infectious complications after transrectal prostate biopsy. Eur Urol.

[60] Loeb S, Vellekoop A, Ahmed HU, Catto J, Emberton M, Nam R, Rosario DJ, Scattoni V, Lotan Y (2013). Systematic review of complications of prostate biopsy. Eur Urol.

[61] Murray KS, Bailey J, Zuk K, Lopez-Corona E, Thrasher JB (2015). A prospective study of erectile function after transrectal ultrasonography-guided prostate biopsy. BJU Int.

[62] Brooks J, Ellis SD, Jones E, Kimminau K, Thrasher JB (2016). Role of social support in prostate cancer treatment decisions: physician perceptions and practices.

[63] Ganz PA, Barry JM, Burke W, Col NF, Corso PS, Dodson E, Hammond ME, Kogan BA, Lynch CF, Newcomer L, Seifter EJ, Tooze JA, Viswanath K, Wessells H (2012). National Institutes of Health state-of-the-science conference: role of active surveillance in the management of men with localized prostate cancer. Ann Intern Med.

[64] Phillips LA, Leventhal EA, Leventhal H (2011). Factors associated with the accuracy of physicians' predictions of patient adherence. Patient Educ Couns.

[65] Zeller A, Taegtmeyer A, Martina B, Battegay E, Tschudi P (2008). Physicians' ability to predict patients' adherence to antihypertensive medication in primary care. Hypertens Res.

[66] Ellis SD, Kimminau K, Jones EV, Petty L, Ellerbeck E, Thrasher JB (2016). Potential barriers to use of prostate cancer treatment decision Aids in urology practice.

[67] Wang EH, Gross CP, Tilburt JC, Yu JB, Nguyen PL, Smaldone MC, Shah ND, Abouassally R, Sun M, Kim SP (2015). Shared decision making and use of decision AIDS for localized prostate cancer : perceptions from radiation oncologists and urologists. JAMA Intern Med.

[68] Makarov DV, Sedlander E, Braithwaite RS, Sherman SE, Zeliadt S, Gross CP, Curnyn C, Shedlin M (2016). A qualitative study to understand guideline-discordant use of imaging to stage incident prostate cancer. Implement Sci.

[69] Saul S (2006). Profit and questions as doctors offer prostate cancer therapy. The New York Times. Vol. December 1, 2006.

[70] Carreyrou J, Tamman M (2010). A device to kill cancer, lift revenue: The wall street journal.

[71] Mitchell JM (2012). Urologists' self-referral for pathology of biopsy specimens linked to increased use and lower prostate cancer detection. Health Aff (Millwood).

[72] Mitchell JM (2013). Urologists’ use of intensity-modulated radiation therapy for prostate cancer. N Engl J Med.

[73] Jacobs BL, Zhang Y, Schroeck FR, Skolarus TA, Wei JT, Montie JE, Gilbert SM, Strope SA, Dunn RL, Miller DC, Hollenbeck BK (2013). Use of advanced treatment technologies among men at low risk of dying from prostate cancer. JAMA.

[74] Elliott SP, Jarosek SL, Wilt TJ, Virnig BA (2010). Reduction in physician reimbursement and use of hormone therapy in prostate cancer. J Natl Cancer Inst.

[75] Shahinian VB, Kuo YF, Gilbert SM (2010). Reimbursement policy and androgen-deprivation therapy for prostate cancer. N Engl J Med.

[76] O'Shaughnessy MJ, Jarosek SL, Virnig BA, Konety BR, Elliott SP (2013). Factors associated with reduction in use of neoadjuvant androgen suppression therapy before radical prostatectomy. Urology.

[77] Shahinian VB, Kuo YF (2015). Reimbursement cuts and changes in urologist use of androgen deprivation therapy for prostate cancer. BMC Urol.

[78] Loeb S, Walter D, Curnyn C, Gold HT, Lepor H, Makarov DV (2016). How active is active surveillance? Intensity of Followup during active surveillance for prostate cancer in the United States. J Urol.

[79] Zeliadt SB, Ramsey SD, Penson DF, Hall IJ, Ekwueme DU, Stroud L, Lee JW (2006). Why do men choose one treatment over another?: a review of patient decision making for localized prostate cancer. Cancer.

[80] Zeliadt SB, Ramsey SD, Van Den Eeden SK, Hamilton AS, Oakley-Girvan I, Penson DF, Fairweather ME, Arora NK, Potosky AL (2010). Patient recruitment methods to evaluate treatment decision making for localized prostate cancer. Am J Clin Oncol.

